# Epidemiology of Undiagnosed Trichomoniasis in a Probability Sample of Urban Young Adults

**DOI:** 10.1371/journal.pone.0090548

**Published:** 2014-03-13

**Authors:** Susan M. Rogers, Charles F. Turner, Marcia Hobbs, William C. Miller, Sylvia Tan, Anthony M. Roman, Elizabeth Eggleston, Maria A. Villarroel, Laxminarayana Ganapathi, James R. Chromy, Emily Erbelding

**Affiliations:** 1 Statistics and Epidemiology, Research Triangle Institute, Washington, District of Columbia, United States of America; 2 Program in Data Analytics and Applied Social Research, City University of New York (Queens College and the Graduate Center), Flushing, New York, United States of America; 3 School of Medicine and Gillings School of Public Health, University of North Carolina, Chapel Hill, North Carolina, United States of America; 4 Center for Survey Research, University of Massachusetts, Boston, Massachusetts, United States of America; 5 Department of Epidemiology, Bloomberg School of Public Health, Johns Hopkins University, Baltimore, Maryland, United States of America; 6 Research Computing Division, Research Triangle Institute, Research Triangle Park, North Carolina, United States of America; 7 Statistics and Epidemiology Division, Research Triangle Institute, Research Triangle Park, North Carolina, United States of America; 8 Division of Infectious Diseases, Johns Hopkins Bayview Medical Center, Baltimore, Maryland, United States of America; State University of Maringá/Universidade Estadual de Maringá, Brazil

## Abstract

*T. vaginalis* infection (trichomoniasis) is the most common curable sexually transmitted infection (STI) in the U.S. It is associated with increased HIV risk and adverse pregnancy outcomes. Trichomoniasis surveillance data do not exist for either national or local populations. The Monitoring STIs Survey Program (MSSP) collected survey data and specimens which were tested using nucleic acid amplification tests to monitor trichomoniasis and other STIs in 2006–09 among a probability sample of young adults (N = 2,936) in Baltimore, Maryland — an urban area with high rates of reported STIs. The estimated prevalence of trichomoniasis was 7.5% (95% CI 6.3, 9.1) in the overall population and 16.1% (95% CI 13.0, 19.8) among Black women. The overwhelming majority of infected men (98.5%) and women (73.3%) were asymptomatic. Infections were more common in both women (OR = 3.6, 95% CI 1.6, 8.2) and men (OR = 9.0, 95% CI 1.8, 44.3) with concurrent chlamydial infection. Trichomoniasis did not vary significantly by age for either men or women. Women with two or more partners in the past year and women with a history of personal or partner incarceration were more likely to have an infection. Overall, these results suggest that routine *T vaginalis* screening in populations at elevated risk of infection should be considered.

## Introduction

*Trichomonas vaginalis* is the most common cause of curable sexually transmitted infection (STI) in the United States [1], [2]. Untreated trichomoniasis is associated with pelvic inflammatory disease, low birth weight, preterm delivery, and increased susceptibility to HIV [3]–[6]. Comorbidity with other STIs is commonly observed [7]–[8]. Despite increasing recognition of the harmful health effects of trichomoniasis [9], many infections go undetected because symptoms are often mild or absent [10]. However, the burden of disease is largely unknown as national surveillance data are not available for trichomoniasis.

Until recently, most studies of trichomoniasis were confined primarily to specialized or clinic populations which do not adequately characterize the incidence or prevalence in the population at large. Clinical detection of trichomoniasis traditionally has relied upon diagnostic techniques with relatively low sensitivity, such as wet mount or culture. The availability of sensitive molecular diagnostics, e.g., transcription mediated amplification (TMA) and polymerase chain reaction (PCR), expanded opportunities for detection of trichomoniasis within broad populations. Using a urine-based PCR, the trichomoniasis prevalence was 2.3% (95% CI 1.8, 2.7) in the 2001–2002 National Longitudinal Study of Adolescent Health (Add Health), a cohort of 18 to 26 year-olds who were U.S. students in grades 7 through 12 in 1994–95 [11]. In the 2001–2004 National Health and Nutrition Examination Survey (NHANES), the prevalence of trichomoniasis was 3.1% (95% CI 2.3, 4.3) of U.S. women aged 14 to 49 years based on PCR of vaginal swabs [12]. Both surveys reported few symptoms among those infected and estimated substantially higher prevalence of infection among Black women (Add Health: 10.5%, 95% CI 8.3, 13.3; NHANES: 13.3%, 95% CI 10.0, 17.7).

While these two surveys provide important data on national prevalence of trichomoniasis, information on the local epidemiology of these infections is sparse. STI epidemics are local phenomena, evolving within communities. National estimates of prevalence obscure variations that arise in subpopulations due to local differences in behavior patterns, sexual networking, screening and case-management. In this paper, we report trichomoniasis prevalence and associated demographic and behavioral characteristics from the 2006–2009 Monitoring STIs Survey Program (MSSP). The MSSP was designed to monitor the epidemiology of sexually transmitted infections across a three-year period among adolescents and young adults residing in Baltimore, Maryland – a metropolitan area with both a historically high incidence of diagnosed STIs based on reports to public health authorities and a high prevalence of undiagnosed STIs based on evidence from past population surveys [13]–[14].

## Methods

### Study sample

The MSSP collected telephone survey data and biospecimens for continuous monitoring of three STIs – gonorrhea, chlamydial infection, and trichomoniasis – among probability samples of adolescents and young adults residing in Baltimore, MD. Recruitment for the MSSP began in September 2006 and ended in June 2009. A stratified, list-assisted, probability sampling design was used to maximize sample efficiency in identifying our target population of English-speaking males and females between 15 and 35 years of age residing in Baltimore households with landline telephones. Over the course of the survey, we estimate that approximately 15% of Baltimore households did not have a landline telephone (see Text S1).

In this design, all households with a landline telephone had a known probability of selection into the sample. Our probability sample included four strata. We sampled the first three strata using commercially-available, regularly updated information on Baltimore households [15]. These strata include (1) households believed to contain someone aged 15–35 years, (2) households with no one aged 15–35, and (3) households with residents of unknown age. The fourth stratum was constructed by selecting all known landline telephone numbers in Baltimore, and removing numbers on the original commercial list. Inclusion of this fourth stratum ensures that the probability sample includes all households with landline telephones, and that each telephone number is in one and only one stratum. Errors in list-sample information (e.g., households that were erroneously thought to have a resident aged 15 to 35) were eliminated during survey screening.

### Survey execution

All sampled households with a known address were sent a lead letter describing the study. Interview staff at the University of Massachusetts, Boston conducted telephone screening and recruitment. Household screening was completed with an adult (18 years of age or older) household member. In screened residences with more than one person aged 15 to 35 years, one member was probabilistically selected (see Text S2).

Minors (<18 years of age) were recruited with parental permission. Parents were informed that their child's survey and test results were confidential and that they would not be shared with parents.

### T-ACASI interview

After obtaining informed consent for the survey interview, interviewers transferred respondents to a T-ACASI system [16]–[17]. T-ACASI increases reporting of sensitive and stigmatized behaviors compared to traditional telephone surveys conducted by human interviewers [18]–[21]. The survey included questions on respondents' demographic characteristics, sexual behaviors, previous STIs, and other health behaviors and took 13 minutes on average to complete. Respondents were compensated $10 to $20 for completing the interview [22].

### Specimen collection

Upon completion of the survey interview, respondents were invited to participate in the STI-testing phase of the study. Participants were informed that they would be re-contacted for a positive gonorrhea or chlamydial infection result and that, as required by law, names and contact information of persons who tested positive would be reported to the local health department. Since at the time the study was initiated the trichomoniasis assay had not been evaluated or cleared by the FDA, participants were informed that they would not be re-contacted regarding their trichomoniasis results [23]–[24]. During year two of the study — following further testing and validation of the test assay by the study's laboratory — the study protocol was amended. During the consent process under the amended protocol, all participants were informed that they would be re-contacted for positive trichomoniasis, gonorrhea, and/or chlamydial infection test result and referred to one of Baltimore's local public health clinics or their own private physician for repeat testing and/or presumptive therapy. (These amended procedures were reviewed and approved by all four IRBs and described in the informed consent documents that were signed by all participants recruited under the amended protocol.)

Participants who agreed to provide a biospecimen for STI testing were mailed a collection kit with instructions, a consent form, and monetary compensation for completing the telephone survey. Most specimens were urine; a small number of women in 2009 (n = 46) provided self-collected vaginal swabs in addition to urine specimens. Specimens were collected in containers with DNA/RNA Protect™ (Sierra Diagnostics, Sonora, CA), designed to prevent nucleic acid degradation for 7–10 days without refrigeration. Participants mailed specimens in pre-addressed postage-paid shipping cartons to the University of North Carolina-Chapel Hill (UNC) Hospitals' McLendon Clinical Laboratories via U.S. Postal Service first class mail. Only specimens submitted with a signed consent form were tested. Participants received $40 to $100 US dollars for mailing in the specimen. (Payments for survey participation and specimens were increased over the course of the study in order to increase the survey response rate and return of specimens for STI testing. The incentive for respondents who agreed to provide a specimen but failed to mail-in their specimen after repeated reminders was increased from $40 to $100 in a final attempt to obtain a specimen. See Text S3 for additional details.)

### Laboratory testing

#### Specimen handling

Urine specimens (2 mL) were transferred to APTIMA Combo 2 Assay urine specimen transport tubes (Gen-Probe, Inc., San Diego, CA) upon receipt at the UNC Hospitals laboratory. *T vaginalis* nucleic acids were detected by transcription-mediated amplification (TMA) using Gen-Probe analyte-specific reagents (TV ASR) using interpretive criteria previously established with vaginal swabs [25]. TV ASR TMA results <10 000 relative light units (RLU) were considered negative, and specimens with ≥30 000 RLU were considered positive. Results from 10 000 to <30 000 RLU were considered equivocal, and specimens were retested. Initially equivocal specimens with repeat test results <10 000 RLU were considered negative; those with repeat results ≥10 000 RLU were considered positive. Trichomoniasis was defined as a repeatedly positive test result. Using these interpretive criteria, TV ASR is 98.2% sensitive and 98.0% specific in latent class analysis compared to wet mount, culture, and a rapid antigen test [25]. The same processed urine specimen was used to detect *N. gonorrhoeae* and *C. trachomatis* nucleic acids using the FDA-cleared APTIMA Combo2 assay (Gen-Probe, Inc., San Diego, CA).

To verify that specimen collection, mailing and processing procedures were acceptable for APTIMA testing, we spiked negative urine specimens (60 mL) in study containers with *T vaginalis* (n = 10) or GC (n = 12) at or near the limits of detection of the assays (10 trichomonads/mL and 250 GC colony-forming units/mL). APTIMA *T vaginalis* ASR results from these spiked specimens and 12 negative control urines that were mailed to the UNC Hospitals laboratory were similar to test results obtained from the same specimens prior to mailing. Quantitative assay outputs in relative light units were not statistically different, and qualitative (negative or positive) results were identical.

### Ethics statement

Participants in the telephone survey provided oral consent. Minors aged 15–17 years were recruited with parental/guardian permission and minor assent; parents were informed that their child's survey and test results were confidential and that they would not be shared with parents. Respondents who agreed to provide a specimen for STI testing were mailed a collection kit (a maximum of three days after the T-ACASI interview) with instructions and a consent form. Only specimens submitted to the laboratory with a signed consent form were tested. All study procedures – including the foregoing consent procedures – were approved by the Institutional Review Boards (IRBs) of Research Triangle Institute, the University of North Carolina at Chapel Hill, the University of Massachusetts-Boston, and the Johns Hopkins Medical Institutions.

### Data Access

The data required to replicate our substantive analyses will be available to authorized researchers under a restricted data use agreement with the Inter-University Consortium for Political and Social Research (ICPSR) at the University of Michigan. Because of the sensitive nature of these data and the risk of deductive disclosure of respondents' identity, researchers must agree to the ICPSR's terms of use which require, in part, compliance with the repository's access regulations and codes of scientific conduct.

### Sample Weighting

Two sets of sample weights were constructed to adjust for the unequal probabilities of selection based on our stratified sample design and survey nonresponse. Initial weights were developed reflecting the inverse probability of selection within each stratum with adjustments for the differing probabilities of selection within households, the number of landline telephones within the household, and survey nonresponse. A post-stratification adjustment was applied to align the sample distribution with the 2006–09 U.S. Census estimates of the Baltimore City population by age, gender, and race/ethnicity [26]. A second set of weights was constructed to compensate for additional differences in the provision of a urine specimen for STI testing among respondents who completed the survey interview.

### Statistical Analyses

Survey estimates of infection prevalence by gender and race/ethnicity were derived using the sample weights described above. Estimated prevalence rates are period rates and represent estimates of infection detected during the 33-month study period. Odds ratios for associations of demographic and behavioral characteristics of respondents with infection status by sex were estimated using logistic regression. Adjusted odds ratios were calculated from multivariable logistic regression models controlling for the effects of race/ethnicity, age, and marital status. Models were estimated separately for men and women. All statistical analyses accounted for the complex sample design of the MSSP using the *svy* algorithms of Stata, version 12 [27].

### Multiple Imputation for Missing Specimens and Survey Data

Multiple imputation models using chained equations (MICE) were used to impute the substantial number of missing TV infection tests (n = 816) and education measurements (n = 777) plus a small number of missing observations for some demographic variables. [The education variable was missing in 777 cases because the question – “highest grade completed” – was not asked (nor was it meaningful) for many respondents under age 20.] Predictor variables used in MICE imputations included age, education, four race-and-gender combinations (Black Female, Black Male, NonBlack Female, and NonBlack Male), STI symptoms in past 3 months (dysuria and discharge), plus 11 other sexual behavior and technical variables. Logit models were used for all imputations except education for which we used an ordered logit model. Using the chained equation multiple imputation procedure we generated 60 sets of imputed data after a burn-in period of 100 iterations.

## Results

### Survey execution

A sample of 73,318 telephone numbers was released over the survey period. 48,136 (65.6%) of these numbers were non-residential (out of service numbers, business telephones, faxes, etc.), 20,435 (27.9%) were residential, and the status of 4,747 (6.5%) numbers was undetermined after repeated attempts. Of the residential numbers, 14,199 (69.5%) were screened and 4998 included one or more eligible household members aged 15 to 35 years. Interviews were completed with 2,936 (58.7%) eligible respondents and 2,136 (72.8%) mailed in specimens for testing.

### Estimated prevalence of trichomoniasis

The estimated prevalence of trichomoniasis was 7.5% (95% CI 6.2, 9.1; Table 1, Left Panel). Accounting for missing specimens with multiple imputation, the prevalence estimate was 7.4% (95% CI 6.0, 8.9), virtually equivalent to that obtained from the tested specimens alone. Similarly equivalent results were obtained for subpopulations defined by race and gender (Table 1, Right Panel). Given these results, our subsequent analyses only present estimates derived from participants providing specimens for testing.

**Table 1:** Estimated prevalence of T. vaginalis infection by race/ethnicity and gender.

	OBSERVED *T. vaginalis*		OBSERVED + IMPUTED *T. vaginalis*	
Race/ethnicity(a)	% (95% CI)	Base N	% (95% CI)	Base N
Black Female	16.1 (13.0, 19.8)	845	16.0 (12.7, 19.2)	1,159
Black Male	5.0 (2.8, 8.8)	454	4.8 (2.1, 7.5)	625
Non-Black Female	4.1 (2.3, 7.0)	477	4.2 (1.9, 6.6)	684
Non-Black Male	0.2 (0.0, 1.0)	344	0.4 (0.0, 1.4)	468
*TOTAL*	7.5 (6.2, 9.1)	2,120	7.4 (6.0, 8.9)	2,936

Results from the 2006–09 Monitoring STIs Survey Program (MSSP). “Observed” estimates use only data from respondents who provided biospecimens for testing. All estimates are weighted to account for differing probabilities of selection and post-stratification adjustment to match Census marginals for Baltimore, Maryland (see text). “Observed + Imputed” estimates include multiply imputed data for respondents with missing biospecimens or other missing data (see text). All base Ns are unweighted. Confidence intervals (CI) were calculated using statistical algorithms that take account of the complex sample design used in the surveys. Confidence intervals for “observed + Imputed” estimates also take account of the impact of imputation of missing data on variance of prevalence estimates.

(a) Persons describing themselves as Hispanic are coded as Non-Black.

### Subpopulation variations in trichomoniasis prevalence

Nearly one in six Black females tested positive for trichomoniasis (16.1%, 95% CI 13.0, 19.8). Estimates were significantly lower among other racial/ethnic and gender subgroups (Table 1). Overall, women were more likely than men to be infected (11.8% vs 2.9%, OR = 4.4, 95% CI 2.4, 8.3) and estimates of infection were significantly higher among Black compared to non-Black men and women in both unadjusted and adjusted analyses (Table 2). Age was not associated with trichomoniasis among men or women when treated as a categorical variable in unadjusted or adjusted analyses.

**Table 2:** Estimated prevalence of T. vaginalis (TV) infection and odds ratios by gender and sociodemographic characteristics.

	FEMALES						MALES						Gender Interaction
Subpopulation:	Tv % (95% CI)	Base N	OR (95% CI)	*p*	AOR (95% CI)	*p*	Tv % (95% CI)	Base N	OR (95% CI)	*p*	AOR (95% CI)	*p*	*P*
**ALL**	11.8 (9.6, 14.3)	1,322	—	—	—	—	2.9 (1.7, 5.1)	798	—	—	—	—	<0.001(c)
**Race**													
Black	16.1 (13.0, 19.8)	845	4.6 (2.4, 8.5)	<0.001	4.4 (2.3, 8.5)	<0.001	5.0 (2.8, 8.8)	454	36.1 (4.6, 281.6)	0.001	26.6 (3.3, 217.4)	0.002	0.059
Non-Black^a^	4.1 (2.3, 7.0)	477	ref		ref		0.2 (0.0, 1.0)	344	Ref		ref		
	*p<0.001*						*p<0.001*						
**Age (in years)**													
15–19	11.4 (7.1, 17.7)	316	1.1 (0.6, 2.1)	>0.50	0.8 (0.4, 1.6)	0.456	3.7 (1.4, 9.8)	260	2.0 (0.4, 9.1)	0.392	0.9 (0.2, 4.2)	>0.50	>0.50
20–24	15.2 (10.8, 20.9)	308	1.5 (0.8, 2.7)	0.163	1.3 (0.7, 2.4)	0.479	3.9 (1.2, 11.7)	152	2.1 (0.4, 10.6)	0.385	1.1 (0.2, 5.7)	>0.50	
25–29	9.3 (6.0, 14.3)	311	0.9 (0.5, 1.7)	>0.50	0.8 (0.4, 1.5)	0.411	2.1 (0.7, 6.6)	190	1.1 (0.2, 5.7)	>0.50	0.8 (0.2, 4.2)	>0.50	
30–35	10.6 (7.1, 15.5)	387	ref		ref		1.9 (0.6, 5.8)	196	Ref		ref		
	*p = 0.323*						*p>0.50*						
**Marital Status**													
Not Married	12.9 (10.4, 15.9)	1,090	ref		ref		3.6 (2.0, 6.3)	650	Ref		ref		0.095
Married	6.3 (3.3, 11.7)	231	0.5 (0.2, 0.9)	0.031	0.7 (0.3, 1.5)	0.335	0.3 (0.0, 1.9)	148	0.1 (0.1, 0.6)	0.012	0.2 (0.0, 1.9)	0.165	
	*p = 0.027*						*p = 0.001*						
**Education** b													
<High school	21.9 (15.1, 30.6)	139	3.3 (1.8, 6.1)	<0.001	2.2 (1.2, 4.3)	0.015	3.5 (1.1, 11.0)	77	3.9 (0.6, 23.8)	0.143	2.6 (0.4, 18.0)	0.337	0.402
High school	14.6 (9.9, 20.9)	253	2.0 (1.1, 3.7)	0.025	1.4 (0.7, 2.5)	0.324	5.2 (2.0, 12.9)	144	5.9 (1.1, 30.9)	0.037	4.3 (0.9, 21.2)	0.070	
>High school	7.8 (5.2, 11.5)	613	ref		ref		0.9 (0.3, 3.4)	317	Ref		ref		
	*p<0.001*						*p = 0.053*						
**Employment** b													
Full-time	9.6 (6.5, 13.8)	557	0.6 (0.3, 1.0)	0.068	0.7 (0.4, 1.2)	0.181	2.3 (1.0, 5.4)	329	2.8 (0.5, 15.4)	0.226	4.4 (0.8, 24.4)	0.091	0.069
Part-time	13.5 (8.5, 20.8)	177	0.9 (0.5, 1.7)	>0.50	0.9 (0.5, 1.8)	>0.50	7.8 (2.1, 25.0)	70	10.0 (1.4, 74.0)	0.024	10.3 (1.4, 77.2)	0.023	
Unemployed	15.2 (10.9, 20.8)	272	ref		ref		0.8 (0.2, 3.5)	138	Ref		ref		
	*p = 0.154*						*p = 0.045*						

Estimates based on respondents who provided urine specimens for testing. Estimates are weighted to account for differing probabilities of selection and post-stratification adjustment to match Census (ACS) marginals for Baltimore, Maryland (see text). Base Ns are unweighted. Odds ratios (ORs) calculated using logistic regression. Gender-specific adjusted ORs (AOR) calculated from multivariable models controlling for race (Black; Non-Black), age (4 categories: 15–19, 20–24, 25–29, 30–35), and marital status (married vs. not married, including widowed, divorced, and separated). Black was omitted in calculation of adjusted ORs for males for education and employment since there were zero TV infections among non-Black respondents in the age range for these variables (20 to 35). P-values shown in column of prevalence estimates are for test of hypothesis that estimates of TV prevalence are independent across categories of the independent variable (e.g., marital status). Variance estimates and confidence intervals (CI) were calculated using statistical algorithms that take account of the complex sample design used in the MSSP surveys. P-values for ORs and adjusted ORs are for test of independence of Tv prevalence across categories of socio-demographic variables taking account of weighting, complex sample design, and covariates (for adjusted ORs).

a Persons describing themselves as Hispanic are coded as Non-Black.

b Ages 20 to 35 only.

c The difference in infection prevalence among men and women is statistically significant (OR = 4.4, 95% CI = 2.4, 8.3, p<0.001).

**Figure 1:**
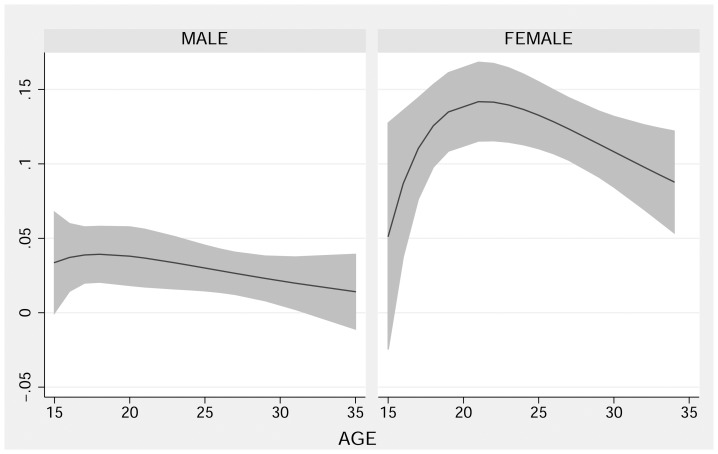
Fractional polynomial fit of age to the probability of undiagnosed infection with *Trichomonas vaginalis* (TV) for men and women. (Predicted TV prevalence and 95% CI calculated using data from 2006–2009 Monitoring STIs Survey Program; see Text S4 for additional details.)

Figure 1 shows the association of trichomoniasis with age using fractional polynomial plots for men and women. No association was observed for males, but females show a seemingly curvilinear relationship between age and infection prevalence that peaks at approximately age 21. This relationship is not, however, statistically significant. Logistic regression models including linear, quadratic, and cubic age variables as predictors of the odds of infection for females had *p* values of 0.256 or higher.

Unmarried men and women were more likely to have trichomoniasis than married persons, but these effects were diminished in adjusted analyses (Table 2). Among women, but not men, having less than a high school education was associated with infection (OR = 3.3, 95% CI 1,8, 6.1) and the association remained after adjusting for race, age, and marital status (adj OR = 2.2, 95% CI 1.2, 4.3). Part-time employment was associated with infection among men (adj OR = 10.3, 95% CI 1.4, 77.2), but there was no association between any employment status and infection prevalence for women (*p* = 0.154).

In considering our results for males, it should be noted that detection of subpopulation variation in trichomoniasis infection among men is compromised by their low rate of observed infection (2.9%, 95% CI 1.7, 5.1). This results in low statistical power and large standard errors for estimates of infection prevalence among subpopulations men.

### Variation by sexual and social behaviors

The number of recent sexual partners was significantly associated with the likelihood of infection among women but not men (Table 3). Women reporting two or more partners in the past year were more likely than women with fewer partners to test positive for trichomoniasis (OR = 2.8, 95% CI 1.8, 4.5). The effect was attenuated somewhat after adjusting for race/ethnicity, age, sex, and marital status but remained statistically significant (adj OR = 2.1, 95% CI 1.3, 3.5). Men and women who reported both opposite and same gender lifetime sex partners were more likely to test positive for trichomoniasis. The effects were weakened after adjusting for race/ethnicity, age, and marital status although the result for females had borderline statistical significance (adj OR = 1.8, 95% CI 1.0, 3.3). The prevalence of trichomoniasis was significantly higher among women (OR = 1.8, 95% CI 1.1, 2.9) and men (OR = 4.7, 95% CI 1.5, 15.2) who reported having a new sex partner in the past three months. After adjustment for demographic characteristics, these effects decreased and were no longer statistically significant for females (adj OR = 1.5, 95% CI 0.9, 2.5) but they were significant for males (adj OR = 3.1, 95% CI 1.1, 9.0).

**Table 3:** Estimated prevalence of T. vaginalis (TV) and odds ratios by gender and sexual behaviors.

	FEMALES						MALES						Gender Interaction
	TV %	Base N	OR (95% CI)^a^	*p*	Adj. OR (95% CI)^a^	*p*	TV (%)	Base N	OR (95% CI)^a^	*p*	Adj. OR (95% CI)^a^	*p*	*p* a
**Lifetime partners include both Males & Females** b													
Yes	19.5%	124	2.0 (1.1, 3.7)	0.030	1.8 (1.0, 3.3)	0.067	9.3%	36	3.8 (0.5, 30.7)	0.210	5.3 (0.6, 45.0)	0.130	>0.50
No	10.9%	1,1198	ref		ref		2.6%	761	ref		ref		
**Had 2+ partners last year** c													
Yes	19.2%	433	2.8 (1.8, 4.5)	<0.001	2.1 (1.3, 3.5)	0.003	4.9%	323	3.3 (1.0, 11.5)	0.060	1.6 (0.5, 5.6)	0.450	>0.500
No	7.8%	888	ref		ref		1.5%	475	ref		ref		
**Had a new partner past 3 months** d													
Yes	17.1%	228	1.8 (1.1, 2.9)	0.029	1.5 (0.9, 2.5)	0.158	7.2%	207	4.7 (1.5, 15.2)	0.009	3.1 (1.1, 9.0)	0.040	0.130
No	10.5%	1,070	ref		ref		1.6%	587	ref		ref		
**Partner had STI in past year** e													
Yes	10.2%	66	0.8 (0.3, 2.2)	>0.50	0.7 (0.3, 1.8)	0.444	8.3%	25	3.2 (0.4, 22.5)	0.247	2.2 (0.3, 15.2)	0.441	0.234
No	11.8%	1,256	ref		ref		2.8%	773	ref		ref		
**Don't know if partner had STI in past year** e													
Yes	13.7%	127	1.2 (0.6, 2.3)	>0.50	1.1 (0.5, 2.2)	>0.50	6.2%	67	2.5 (0.4, 13.8)	0.305	1.7 (0.3, 10.7)	>0.50	0.453
No	11.5%	1,195	ref		ref		2.6%	731	ref		ref		
**Ever forced to have sex** f													
Yes	17.5%	206	1.7 (1.0, 3.0)	0.038	1.8 (1.1, 3.1)	0.028	0.0%	18	(h)		(h)		(h)
No	10.8%	1,111	ref		ref		3.0%	766					
**Respondent or partner ever incarcerated** g													
Yes	17.8%	527	2.7 (1.7, 4.3)	<0.001	2.0 (1.2, 3.4)	0.012	4.5%	218	2.0 (0.6, 6.2)	0.24	1.3 (0.5, 3.7)	>0.50	>0.500
No	7.5%	793	ref		ref		2.3%	578	ref		ref		

a Odds ratios (ORs) calculated using logistic regression. Gender-specific adjusted ORs (AORs) calculated from multivariable models controlling for race (Black; Non-Black), age (4 categories: 15–19, 20–24, 25–29, 30–35), and marital status (married vs. not married, including widowed, divorced, and separated). Gender Interaction tests equivalence of unadjusted ORs for males and females (i.e., logistic regression models odds of Tv as a function of Female, Behavioral variable, and Female-by-Behavioral variable).

b Referent group includes respondents who reported solely either male or female lifetime sexual partners. Respondents with no lifetime partners recoded as ‘no’.

c Referent group includes respondents reporting less than 2 partners in the past year. Respondents with no lifetime partners recoded to no partners last year. (5 respondents tested positive for TV but reported zero lifetime partners.)

d Respondents reporting no lifetime partners or no partners in the past year recoded to no new partners in the past 3 months.

e Referent group includes respondents who reported knowing whether or not their partner(s) in the past year had an STI. Respondents with no lifetime partners or no partners in the past year recoded as “knowing”.

f Referent group includes respondents who reported never being forced to have sex with someone when they didn't want to. Respondents with no lifetime partners recoded as never having forced sex.

g Respondents with no lifetime partners recoded as never having partners who were incarcerated.

h ORs not calculated because none of the 32 men who reported forced sex also tested positive for Tv.

Women, but not men, with a history of personal or partner incarceration were more likely to have trichomoniasis (OR = 2.7, 95% CI 1.7, 4.3) and the association remained significant after adjustment (adj OR = 2.0, 95% CI = 1.2, 3.4). A formal test for gender interaction does not reject the null hypothesis that the pattern of association between incarceration and infection prevalence was equivalent for males and female (*p*>0.50).

Women with a self-reported history of STIs (OR = 1.9, 95% CI 1.2, 3.0) or a previous diagnosis with trichomoniasis (OR = 2.1, 95% CI 1.2, 3.5) were more likely to have trichomoniasis although the effects did not persist after adjustment (Table 4). Infection with trichomoniasis was not associated with recent antibiotic use in our sample, although men who reported a health care visit in the past three months were significantly less likely to test positive for trichomoniasis (adj OR = 0.3, 95% CI 0.1, 1.0).

**Table 4:** Estimated prevalence of T. vaginalis (Tv) and odds ratios by gender and health behaviors.

	FEMALES						MALES						Gender Interaction
	Base N	Tv %	OR (95% CI)^a^	*p*	Adj. OR (95% CI)^a^	*p*	Base N	Tv (%)	OR (95% CI)^a^	*p*	Adj. OR (95% CI)^a^	*p*	*p* a
**Previously had an STI** b													
Yes	411	16.8%	1.9 (1.2, 3.0)	0.006	1.4 (0.8, 2.3)	0.254	92	2.5%	0.9 (0.2, 3.7)	>0.50	0.5 (0.1, 1.9)	0.283	0.309
No	910	9.6%	ref		ref		706	3.0%	ref		ref		
**Previously had ** ***T. vaginalis*** c													
Yes	185	19.7%	2.1 (1.2, 3.5)	0.005	1.6 (0.9, 2.9)	0.094	16	0.0%	(e)		(e)		(e)
No	1,136	10.5%	ref		ref		782	3.0%					
**Current chlamydial infection**													
Yes	44	31.0%	3.6 (1.6, 8.2)	0.002	2.8 (1.2, 6.3)	0.013	26	17.1%	9.0 (1.8, 44.3)	0.007	5.3 (1.1, 26.6)	0.043	0.319
No	1,278	11.1%	ref		ref		772	2.3%	ref		ref		
**Symptoms in past 3 months** d													
Yes	224	19.7%	2.2 (1.3, 3.7)	0.005	2.0 (1.2, 3.5)	0.012	49	0.8%	0.2 (0.0, 2.0)	0.189	0.3 (0.0, 2.3)	0.237	0.049
No	1,097	10.2%	ref		ref		749	3.0%	ref		ref		
**Doctor or clinic visit in past 3 months**													
Yes	870	11.6%	1.0 (0.6, 1.5)	>0.50	0.9 (0.5, 1.4)	0.499	367	1.4%	0.3 (0.1, 1.1)	0.081	0.3 (0.1, 1.0)	0.051	0.119
No	447	12.1%	ref		ref		429	4.1%	ref		ref		
**Antibiotic use in past month**													
Yes	209	12.7%	1.1 (0.6, 1.9)	>0.50	1.0 (0.5, 1.7)	>0.50	93	1.4%	0.5 (0.1, 2.6)	0.427	0.6 (0.1, 3.0)	>0.50	0.388
No	1,112	11.6%	ref		ref		702	2.7%	ref		ref		

a Odds ratios (ORs) calculated using logistic regression. Gender-specific adjusted ORs (AORs) calculated from multivariable models controlling for race (Black; Non-Black), age (4 categories: 15–19, 20–24, 25–29, 30–35), and marital status (married vs. not married, including widowed, divorced, and separated). Gender Interaction tests equivalence of unadjusted ORs for males and females (i.e., logistic regression models odds of Tv as a function of Female, Behavioral variable, and Female-by-Behavioral variable).

b Previous STI includes self-reported diagnoses of C. trachomatis, N. gonorrhea, and/or T. vaginalis.

c Self-reported previous diagnosis of T. vaginalis infection.

d Self-reported symptoms of dysuria and/or discharge.

e ORs not calculated because none of the 16 men with a prior diagnosis of Tv also tested positive for Tv in the survey.

### Co-morbidity with chlamydial infection

Both women and men who had a current chlamydial infection were much more likely to also have trichomoniasis. Among women, 31% of those with chlamydial infection also had a trichomoniasis infection compared to 11.1% of women with no chlamydial infection (OR = 3.6, 95% CI 1.6, 8.2). Similarly for men, 17.1% of those with an undiagnosed chlamydial infection also had undiagnosed trichomoniasis compared to 2.3% of other men (OR = 9.0, 95% CI 1.8, 44.3). These effects persisted after adjusting for race, age, and marital status (adj OR = 2.8, 95% CI 1.2, 6.3 for women, and OR = 5.3, 95% CI 1.1, 26.6 for men).

### Gender Interactions

We had anticipated that there would be many associations between infection prevalence and respondents' characteristics that would vary significantly by gender. However, none of the tests for gender by sociodemographic variable interaction were significant at the 0.05 level (Table 2). We also found only one significant gender interaction for respondent sexual or health behavioral characteristics (*p* = 0.049) — for presence of symptoms in the past 3 months (Table 4). Women reporting symptoms in the past three months were more likely than other women to have trichomoniasis (19.7% vs. 10.2%, adj OR = 2.0, 95% CI 1.2, 3.5) while for men the reverse was true but not statistically significant (0.8% vs. 3.0%, adj OR = 0.3, 95% CI 0.0, 2.3).

### Symptomatic and asymptomatic trichomoniasis

Only one of 18 men (unweighted) with trichomoniasis reported symptoms in the past three months. Most men (94.2%) and women (84.1%) reported neither discharge nor dysuria in the three months prior to the survey (Table 5). Furthermore, most participants with undiagnosed infections were asymptomatic. Of persons with trichomoniasis, 98.5% of men (95% CI: 89.1, 99.8) and 73.3% of women (95% CI 62.6, 82.0) reported neither dysuria nor discharge in the three months prior to the survey.

**Table 5:** Presence and absence of reported symptoms by gender and infection with trichomoniasis.

	SYMPTOMS^a^		NO SYMPTOMS^a^	
GENDER and INFECTION STATUS	% (95% CI)	Base N	% (95% CI)	Base N
**WOMEN**	15.9 (13.6, 18.5)	224	84.1 (81.5, 86.4)	1,097
With trichomoniasis	26.7 (18.1, 37.4)	37	73.3 (62.6, 82.0)	101
No trichomoniasis	14.4 (12.1, 17.0)	187	85.6 (82.9, 87.8)	996
**MEN**	5.8 (4.2, 8.1)	49	94.2 (92.0, 95.8)	749
With trichomoniasis	1.6 (0.0, 10.9)	1	98.5 (89.1, 99.8)	17
No trichomoniasis	6.0 (4.3, 8.3)	48	94.1 (91.7, 95.8)	732

All estimates are weighted to account for differing probabilities of selection and post-stratification adjustment to match Census (ACS) marginals for Baltimore, Maryland (see text); all base Ns are unweighted. Confidence intervals (CI) were calculated using statistical algorithms that take account of the complex sample design used in the MSSP surveys.

a Symptoms are dysuria (burning on urination) and discharge in the three months preceding the survey. Subjects were coded positive for symptoms if they reported either or both symptoms.

## Discussion

The MSSP was designed to monitor the prevalence of trichomoniasis and other STIs in a population typically identified as being at high risk of sexually transmitted infections – young adults residing in an urban center with high rates of diagnosed STIs and HIV. In this population, female gender, Black race, and having less than a high school education were significantly associated with likelihood of trichomoniasis. Estimates of infection were particularly troublesome among Black women. Our results suggest that routine screening should be considered to prevent the morbidity associated with trichomoniasis in populations at elevated risk of infection, including sexually active young women in Baltimore and similar venues [1], [28]–[30].

Trichomoniasis poses two major difficulties for public health strategies that seek to contain the disease in the general population. First, unlike other STIs such as chlamydial infection, trichomoniasis was not consistently observed in a particular age group. We found no association between age and infection prevalence across the age range 15 to 35 for either women or men. Second, the overwhelming majority of infections were asymptomatic and symptoms were common among uninfected persons. The latter finding renders self-referral for treatment ineffective as a strategy for controlling trichomoniasis in the general population. Population screening is a logical alternative, but the lack of an association with age in the age group studied implies that screening may not be effectively targeted on a narrow age range, e.g., adolescents and young adults.

Among women, multiple sexual partners, experiences of forced sex, a history of self or partner incarceration, and concomitant chlamydial infection were risk markers for prevalent trichomoniasis. Among men, current chlamydial infection and new sex partners remained significantly associated with trichomoniasis in multivariable analyses while recent doctor or clinic visits were associated with a lower prevalence of infection. (Smaller sample sizes may have limited our ability to detect associations with other characteristics among men.)

These results are consistent with findings from other large-scale population-based surveys. The Wave III Add Health study observed substantial overlap of trichomoniasis with chlamydial infection, although the effects were most pronounced among women [11]. Among women in the 2001–04 NHANES, prevalence of chlamydial infection was higher among women under 26 years of age with trichomoniasis. They observed no differences among older women.

### Study Limitations

Our results should be interpreted with awareness of the limitations of our research. *The first limitation* arises from the locale of our research. This research studied the adolescent and young adult population of the city of Baltimore, MD. Numerous studies have demonstrated that Baltimore city has a high incidence of both diagnosed STIs [31] and undiagnosed STIs that persist in this population [14]. While results from this venue may generalize to other urban locales with similar characteristics, it is unlikely that our findings will generalize to non-urban populations with a low incidence of diagnosed STIs and a low prevalence of undiagnosed STIs.

*The second limitation* arises from the substantial level of non-response to the telephone survey including a greater proportion of females than males who responded to the survey and to the subsequent request for biospecimens for STI assay. Following typical survey practice, we used poststratification weights to align our survey samples to the demographic composition of the Baltimore population, and we employed covariate adjustments for gender, age, race, and marital status to provide further control in our statistical analyses. It should be recognized, however, that neither poststratification weights nor the covariate adjustments employed in our statistical analyses can guarantee sample equivalence.

The non-response problem in telephone surveys is a well recognized and growing problem. The MSSP did much better than many telephone surveys in obtaining household and respondent cooperation. (Survey screening was completed with 69.5% of households identified as residential, and interviews were completed with 58.7% of screened respondents who were identified as eligible for interview.) While MSSP may have done better than the average telephone survey [32], there is ample room for concern that non-response bias may distort our results. The available evidence suggests that the impact of this non-response may be less substantial than one might fear [32], [33]. However, we cannot know with certainty what impact this non-response would have for the findings of the MSSP.

*The third limitation* arises from the fact that only 2,136 of 2,936 (72.8%) of survey respondents provided biospecimens for STI testing. Multiple imputation suggests that this biospecimen non-response had a trivial impact on STI prevalence (see Table 1). A similar conclusion was reached regarding STI biospecimen non-response in a national survey of adolescent males [34] and an earlier in-person study of Baltimore adults [14]. We cannot, nonetheless, be certain that biospecimen non-response does not affect our conclusions.

### Conclusion

Data from the MSSP confirm findings from other population-based and clinical studies that suggest trichomoniasis is highly prevalent in the general population ---- particularly among Black women. Vigorous public health interventions to reduce this prevalence and to potentially avoid the complications associated with infection are clearly warranted. The MSSP research model and similar population survey programs provide a much-needed paradigm for monitoring the impact of such interventions on the prevalence of untreated and largely asymptomatic infections that persist in the population, both nationally and locally. A key challenge for future research will be adapting this paradigm so that it can be implemented at a cost that is affordable to local public health departments.

## Supporting Information

Text S1 (DOC)

Text S2 (DOC)

Text S3 (DOC)

Text S4 (DOC)
