# Supplementary material for: Epidemiology of Undiagnosed Trichomoniasis in a Probability Sample of Urban Young Adults
Source: PLoS One. 2014 Mar 13;9(3):e90548. doi: 10.1371/journal.pone.0090548 (PMC3953116; doi:10.1371/journal.pone.0090548)
Supplement: Text S1 — (DOC) [file pone.0090548.s001.doc]

**SUPPLEMENTAL MATERIALS**

Text S1

We derived this composite estimate using the National Health Interview Survey’s (NHIS) finding [1] that the proportion of Baltimore Households that lacked a landline telephone was 14.1% in 2007, 15.2% in 2008, and 23.1% in 2009. (The NHIS is an in-person federal survey conducted for CDC by the Bureau of the Census with annual national samples of approximately 35,000 households; see www.cdc.gov/nchs/nhis/about_nhis.htm#sample_design.) For our composite estimate, we conservatively assumed that the unknown percent of Baltimore households without landline phone service in 2006 was as high as it was in 2007 (14.1%). We then calculated a weighted average adjusting for the relative number of specimens collected in each year of the survey.

1. Blumberg SJ, Luke JV, Ganesh N, Davern ME, Boudreaux MH et al (2011) Wireless substitution: state-level estimates from the National Health Interview Survey, January 2007-June 2010. Natl Health Stat Report Apr 20;(39):1-26, 28.
