# Supplementary material for: Epidemiology of Undiagnosed Trichomoniasis in a Probability Sample of Urban Young Adults
Source: PLoS One. 2014 Mar 13;9(3):e90548. doi: 10.1371/journal.pone.0090548 (PMC3953116; doi:10.1371/journal.pone.0090548)
Supplement: Text S2 — (DOC) [file pone.0090548.s002.doc]

**SUPPLEMENTAL MATERIALS**

Text S2

At the outset of the survey, each eligible household member was given an equal chance of being selected as the participant in this survey. Since for this age group (15-35), getting people on the telephone is a time consuming and costly task, a new respondent selection model was adopted six months into the study. This model gave an increased probability to selecting the person on the telephone answering the screening questions if that person was an eligible respondent. The procedure worked in the following manner. If the person answering the screening questions was not eligible, then as before, all eligible people in the household were given an equal chance of selection, namely (1/n) where n is the number of eligible people in the household. If the person answering the screening questions was found to be eligible, then that person was assigned a (2/(n+1)) probability of selection with all other eligible people in the household getting probabilities of (1/(n+1)) of being selected. This change was made to increase efficiency, reduce cost, and still maintain a probabilistic method of within household respondent selection. A complete description of this change and the effects of this change are found in Roman et.al [22].
