# Supplementary material for: Epidemiology of Undiagnosed Trichomoniasis in a Probability Sample of Urban Young Adults
Source: PLoS One. 2014 Mar 13;9(3):e90548. doi: 10.1371/journal.pone.0090548 (PMC3953116; doi:10.1371/journal.pone.0090548)
Supplement: Text S3 — (DOC) [file pone.0090548.s003.doc]

**SUPPLEMENTAL MATERIALS**

Text S3

Specimen kits were mailed to all respondents who agreed to receive them within three days of the date they agreed. If a specimen was not received by the lab within two weeks of the date it was mailed, the respondent was called in an attempt to get them to send the specimen to the lab. After three unsuccessful call attempts, a letter was sent to the respondent asking them to send the specimen to the lab as they agreed. In this letter, the amount paid for a return specimen was raised from $40 to $100 as a last attempt to increase the return rate of the biospecimen.
