# Supplementary material for: Epidemiology of Undiagnosed Trichomoniasis in a Probability Sample of Urban Young Adults
Source: PLoS One. 2014 Mar 13;9(3):e90548. doi: 10.1371/journal.pone.0090548 (PMC3953116; doi:10.1371/journal.pone.0090548)
Supplement: Text S4 — (DOC) [file pone.0090548.s004.doc]

**SUPPLEMENTAL MATERIALS**

Text S4

Sample data are weighted but the software (Stata version 12) used to plot the predicted values and the confidence intervals could not account for the impact of stratification on the precision of the predicted values.  Software that allows the introduction of a stratification effect (SAS SURVEYLOGISTIC) was used to estimate the impact of incorporating stratification into the estimation process.  A similar fractional model was utilized and the width of the confidence intervals was evaluated with and without accounting for stratification.  As expected, stratification reduced the standard errors of the predicted values, but the reduction was less than a tenth of a percent; for the model applied to females, the average width of the confidence interval over the range of ages from 15 to 35 decreased from 0.122445 to 0.122324.  While some caution in interpreting Figure 1 confidence bounds may be warranted, the impact of ignoring the stratification effect is truly negligible from a substantive viewpoint.
